# Observing the silent world under COVID-19 with a comprehensive impact analysis based on human mobility

**DOI:** 10.1038/s41598-021-94060-4

**Published:** 2021-07-19

**Authors:** Shaobin Wang, Yun Tong, Yupeng Fan, Haimeng Liu, Jun Wu, Zheye Wang, Chuanglin Fang

**Affiliations:** 1 Institute of Geographic Sciences and Natural Resources Research, Chinese Academy of Sciences, Beijing, China; 2 School of Tourism, Hainan University, Haikou, China; 3 Program in Public Health, Susan and Henry Samueli College of Health Sciences, University of California, Irvine, USA; 4 Kinder Institute for Urban Research, Rice University, Houston, USA

**Keywords:** Sustainability, Environmental impact, Socioeconomic scenarios

## Abstract

Since spring 2020, the human world seems to be exceptionally silent due to mobility reduction caused by the COVID-19 pandemic. To better measure the real-time decline of human mobility and changes in socio-economic activities in a timely manner, we constructed a silent index (SI) based on Google’s mobility data. We systematically investigated the relations between SI, new COVID-19 cases, government policy, and the level of economic development. Results showed a drastic impact of the COVID-19 pandemic on increasing SI. The impact of COVID-19 on human mobility varied significantly by country and place. Bi-directional dynamic relationships between SI and the new COVID-19 cases were detected, with a lagging period of one to two weeks. The travel restriction and social policies could immediately affect SI in one week; however, could not effectively sustain in the long run. SI may reflect the disturbing impact of disasters or catastrophic events on the activities related to the global or national economy. Underdeveloped countries are more affected by the COVID-19 pandemic.

## Introduction

The novel Coronavirus (COVID-19) pandemic is a global threat with escalating health and social-economic challenges^1,2^. From spring 2020, the human world has been extremely silent during the COVID-19 pandemic due to several measures practiced globally to slow down the spread of COVID-19, e.g., social distancing, teleworking, distance learning, banning or reducing crowds, closing non-essential facilities and services, and staying at home orders. Meanwhile, Internet use has increased rapidly to compensate for the reduced face-to-face interactions^3^. Although these non-pharmaceutical interventions effectively reduced the spread of the virus^4,5^, the massive lockdowns and reduction in human mobility have inadvertently affected the global economy for business, transport, manufacturing, tourism, entertainment, and restaurants^6–9^. It also had great impacts on gender equality^10^, education^11^, and global poverty^12^. Population-level human mobility data are becoming increasingly available from location-based services and mobile phone applications^13,14^. During the COVID-19 crisis, some companies such as Google, Apple, and Facebook publish real-time daily mobility data to study human movement trends over time^15,16^.

Accordingly, the use of mobility data has gained emerging interest in studying the impact of the COVID-19 pandemic. A large reduction in mobility has been detected globally since the onset of the COVID-19 threat and administrative restrictions on human interactions^17,18^. Meanwhile, real-time mobility data helped elucidate the COVID-19 transmission and ascertain the implementation of strict control measures, which substantially mitigated the spread of COVID-19^19–21^. Based on mobile phone location data, many studies found that reduced population mobility during the pandemic slowed down the spread of infections^22–24^. Public policies leading to decreased human mobility were associated with lower COVID-19 cases and deaths^25^ and reduced COVID-19 spread^26^. Similar results were observed across many countries globally^27,28^. However, most data published on human activity behavior have focused exclusively on a single country, especially in the United States and Europe^26,29,30^. Meanwhile, few studies have systematically quantified the different relationships between mobility, COVID-19 cases, and government interventions across countries. Moreover, some studies have shown that human mobility is strongly associated with regional socio-economic indicators such as per capita income, poverty rate, unemployment, and education^31–36^. However, these studies are limited in numbers and focus on the socioeconomic status of a person or family. There is remarkably little work on evaluating the socio-economic impact of COVID-19 based on human mobility at the country scale.

Therefore, in this work, we aim to estimate the global impact of COVID-19 based on human mobility and to investigate the relations between mobility, new COVID-19 cases, and government policy across countries over time. The heterogeneity of the relations by place categories and countries is also discussed. In this study, we constructed a silent index (SI) across countries based on the Google Community Mobility Dataset, an aggregate of the place-based activity behavior of millions of individuals in various countries through their location-enabled mobile device data. The SI was constructed based on five-place categories (retail & recreation, grocery & pharmacy, transit stations, workplaces, and park), and as a global index by weighting the country-level values by the total population of each country. The SI was used to comprehensively quantify the daily changes in human mobility and served as a proxy indicator of change in socio-economic activities (see Methods). We examined the potential relations between SI, new COVID-19 cases, and government policy using Panel vector autoregression (PVAR) and Impulse response function (IRF). We demonstrate that a large-scale and holistic picture of the global shock of COVID-19 can be obtained based on the real-time daily mobility data with fine temporal granularity.

## Results

### How silent the world was in 2020 globally, nationally and at specific places?

First, we constructed the silent index (SI) framework to assess the variation of human mobility at the county level based on Google's mobility big data. The name of this index is inspired by *Silent Spring*, which described the absence of the sound of birds and insects due to the overutilization of pesticides in the environment. The SI was calculated based on the Google Community Mobility Dataset in five categorized places, and this index can describe the relative change of daily human mobility compared with that before the COVID-19 pandemic for a specific region.

**Figure 1:**
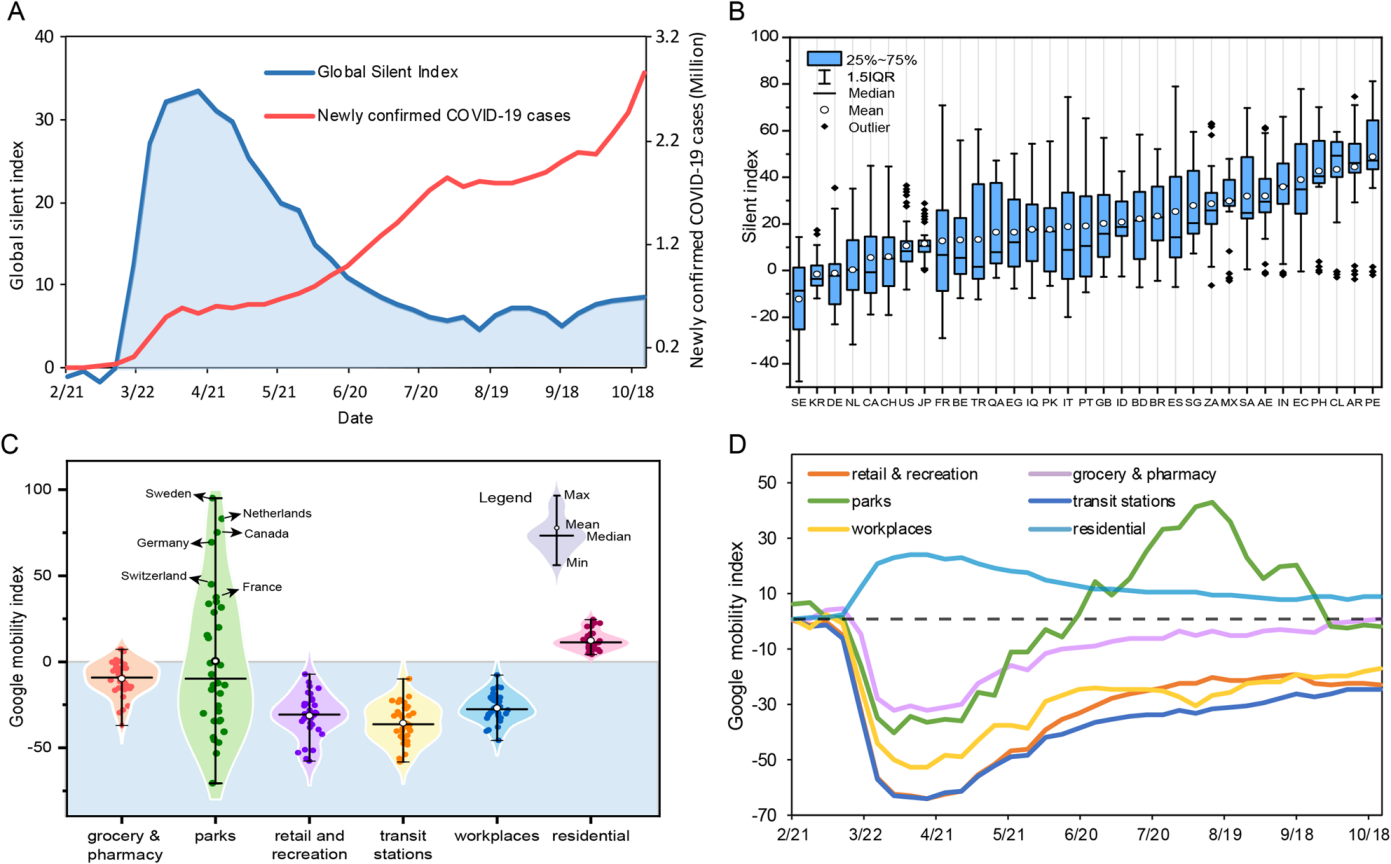
Variation of the silent world globally, nationally and by place. (**A**) Temporal variation of global SI and the number of newly confirmed COVID-19 cases from 126 countries from the baseline day to middle October 2020. (**B**) Box-plot of SI since the 100 confirmed COVID-19 cases reported in 33 selected countries. (**C**) Violin-plot of Google mobility index (see Methods) by six place categories in 33 countries. (**D**) Temporal variation of mobility in six places in 33 countries.

Then, we analyzed the global SI from before the start of the global COVID-19 pandemic in February 2020 to mid-October 2020. Figure 1 compares the number of newly confirmed COVID-19 cases (red curve) and the global SI (see Methods) based on weekly data from 126 countries since February 15th, 2020. The global SI rose rapidly right after the World Health Organization (WHO) announced a pandemic phase on March 11^th^, 2020, with a peak value of 33 in mid-April, followed by a consistent decline till August 2020. The average global SI is 12.5 during this period. In comparison, the number of newly confirmed daily COVID-19 cases increased from March till October 2020 (Fig. 1A).

Thirty-three countries were selected to closely examine the temporal variation of SI across countries. These countries had high popularity of Google service on mobile phones and experienced a relatively serious COVID-19 pandemic. Figure 1B shows that the SI values varied greatly by country during the pandemic, with the lowest value of -12.29 in Sweden and the highest value of 48.75 in Peru. The average SI values were negative in three countries (i.e., Sweden, Germany, and South Korea), and positive in other countries, indicating reduced human mobility in these countries compared with that in the baseline period before the pandemic (See Methods for details of a baseline period). In addition, the range and variation of SI differed greatly among the 33 countries (Fig. 1B). Spain, Ecuador, France, and Italy had the larger range of SI values, while some other countries including the United States, Japan, South Africa, Mexico, Chile, Peru, Argentina, and the Philippines had some sporadic extreme values (mostly toward the lower end with some negative values), indicating large fluctuations.

The human mobility in specific places showed significant differences in 33 selected countries, with the largest difference seen for the park and the smallest difference for residences (Fig. 1C). The average human mobility by place category ranked as: residence > grocery & pharmacy > parks > workplaces > retail & recreation > transit stations. The residential mobility values in all countries were positive. As one might expect, people are forced to stay at home, which results in higher mobility in residential places during the COVID-19 pandemic. Most mobility values in other place categories were negative except positive values for parks (more park visits) in several countries, such as Sweden, Netherlands, Canada, Germany, Switzerland, and France.

Compared to the pre- COVID-19 period, retail & recreation areas, transit stations, and workplaces became the least mobile. Entertainment is the easiest for people to give away. It is also relatively easy to change to online shopping and telecommuting or work from home. Grocery and pharmacy are places that meet people’s essential needs thus have less reduced mobility. Parks had the widest range of Google mobility index values, with some countries having increased park visits during the pandemic. In addition, the mobility index of grocery & pharmacy, workplaces, retail & recreation, and transit stations showed a similar trend, with a sharp decline starting in late March till April followed by a gradual increase, but never reaching the pre-pandemic levels at the end of the study period. The mobility of parks showed a rebound from June to September, 2020, far exceeding the pre-pandemic level after the reduction in April to May, which may reflect a strong demand for natural contact after long-term stay-home orders and also more outdoor activities in the warmer summertime (Fig. 1D).

### Temporal variations of SI and new COVID-19 cases

**Figure 2:**
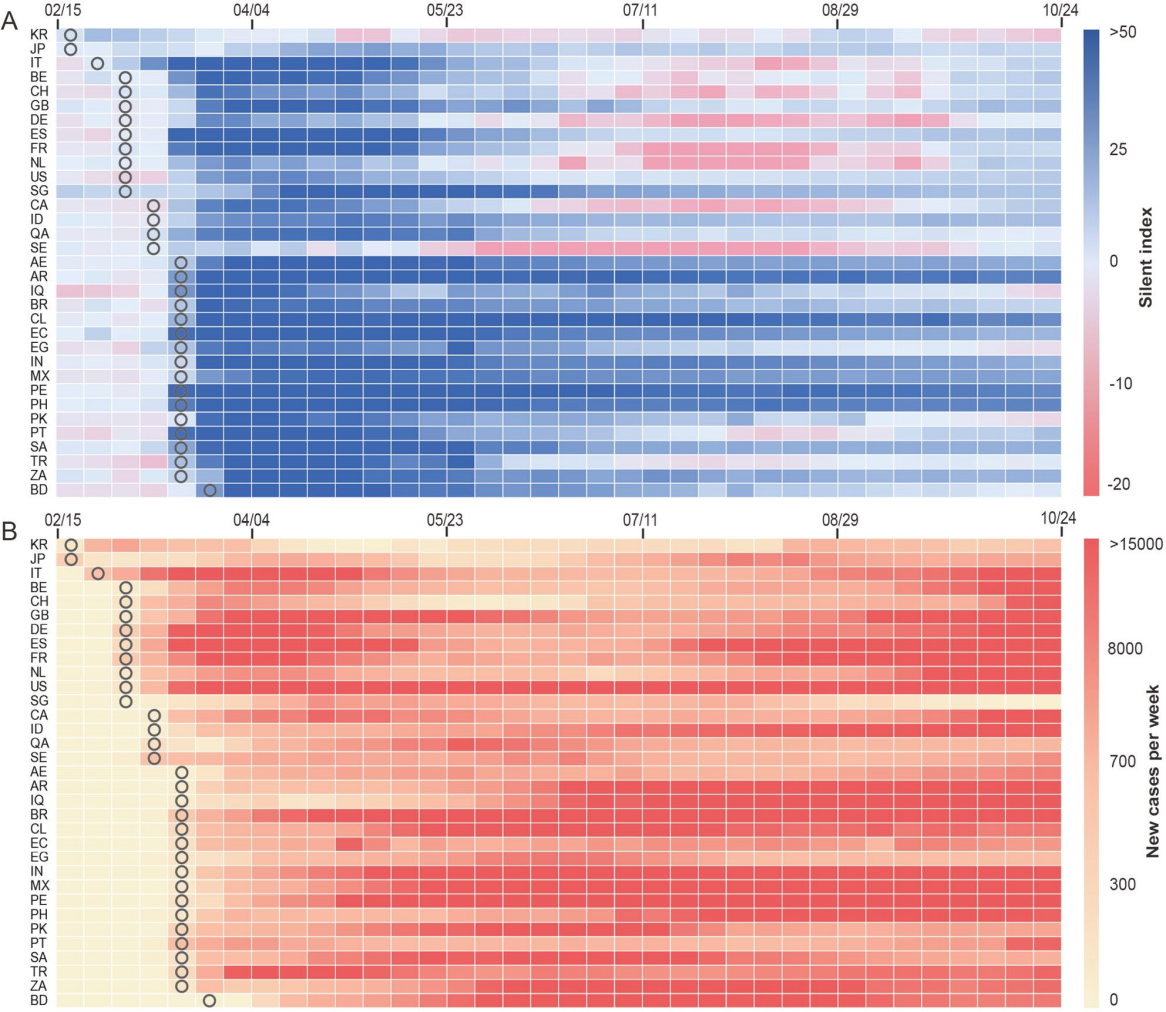
Temporal variations of SI and new COVID-19 cases. The changing of SI (**A**) and newly confirmed weekly cases of COVID-19 (**B**) in 33 selected countries from the baseline day to late October 2020 were visualized with a heatmap. The symbol “○” indicated the week when the cumulative number of COVID-19 cases reached 100. The 33 countries’ full names can be seen in Table S2.

Although most of the 33 countries experienced higher SI during the COVID-19 outbreak compared to the period before the outbreak especially from March to May 2020, the magnitude of changes in SI varied across countries and over time (Fig. 2A). First, the SI was mostly negative before the date when cumulative COVID-19 cases reached 100, indicating that human mobility slightly increased compared to that in the baseline period. The SI became positive in most countries, especially in Ecuador, Peru, Philippines, Portugal, et al., after the WHO announced the global pandemic of COVID-19 on March 11^th^. The result indicates the important role of the WHO announcement in providing guidance for individual countries to develop containment and closure policies and fast responses that resulted in reduced population movement and contact in the early stage of the outbreak. Second, the difference among countries became less pronounced after more countries implemented containment and control measures from early March to May 2020. However, the SI decreased sharply in July–September and then rebounded in October in many European countries including Belgium, Switzerland, Germany, France, Italy, Netherlands, etc. after experiencing high SI values in April and May. This is related to the temporal variations of new COVID-19 cases in each country (Fig. 2B). In addition, the SI of South Korea and Sweden stayed around 0 and changed little throughout the study period, indicating the little impact of the COVID-19 pandemic on population mobility.

### Relationship between SI, COVID-19 cases, and government policy

**Figure 3:**
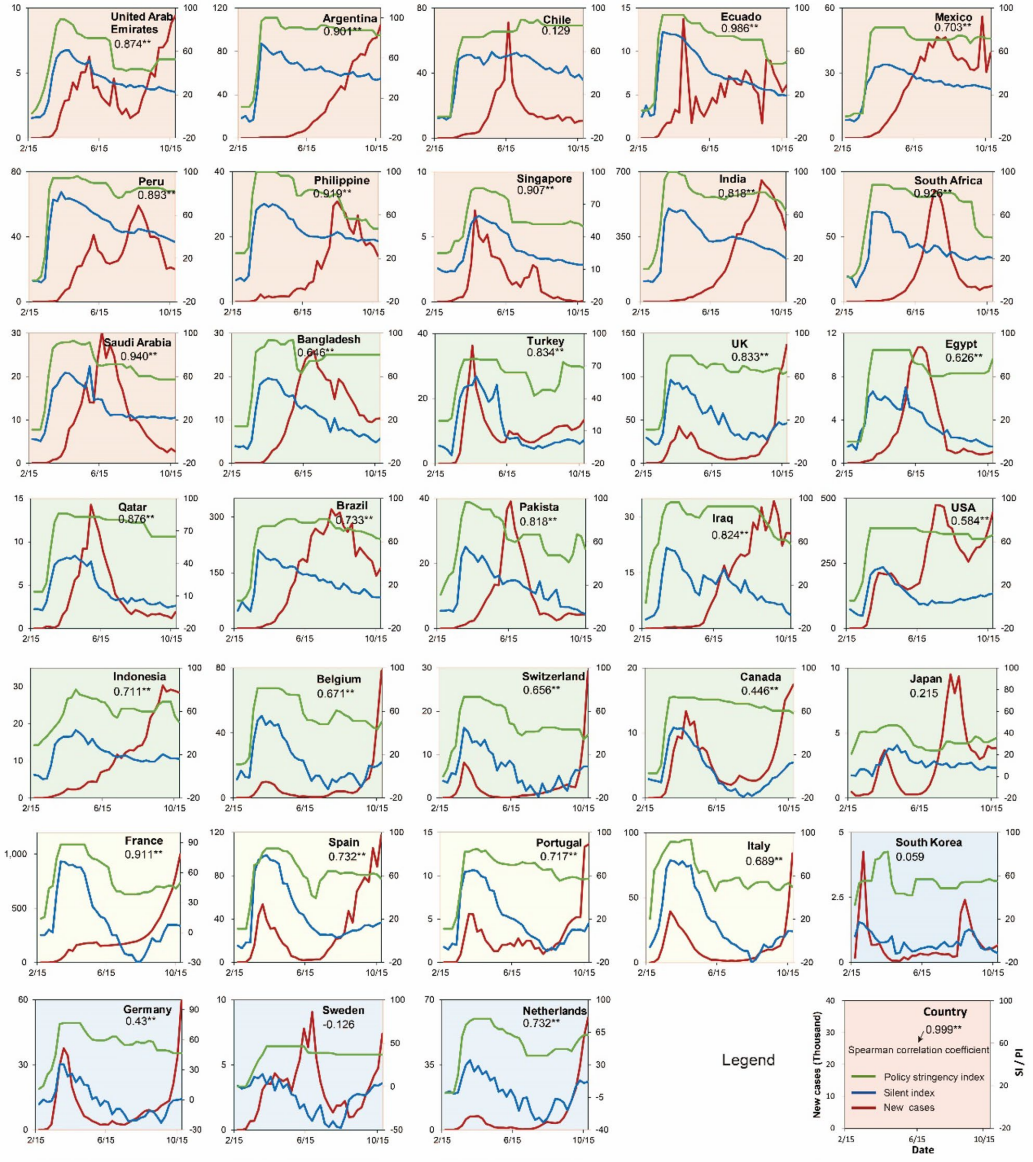
Temporal variation of SI, the new COVID-19 cases, and policy stringency index by country. Four groups across the countries are clustered with different colors by dendrogram in the heatmap of SI (see Fig. S1 and Table S1). Spearman correlations between SI and PI are labeled for each country, and **Means a significant correlation at the 0.01 level.

The government response measures play a critical role in stemming the infection of COVID-19^37^. Government interventions to the COVID-19 spread were measured by policy stringency index (PI)^38^. Figure 3 illustrated the temporal variation of SI, the new COVID-19 cases, and the policy stringency index (PI, see Methods) by country during the study period. Based on the hierarchical clustering of the heatmap of SI variations, we identified four clusters (see Fig. S1). The first cluster showed a rapid increase of SI in early March, followed by a gentle decline trend afterward with no large fluctuations. The second cluster showed a steep rise of SI in early March and a quick decline trend afterward. The third cluster presented a similarly steep rise as the second cluster, but the peak of SI was higher than 60 and declined soon after reaching the peak values; this cluster had the largest range of SI values. The fourth cluster had the lowest SI values with small fluctuation.

Significant Spearman correlations between SI and PI were identified in 29 out of 33 countries (Fig. 3). These 29 countries also experienced an earlier rise in the PI curve than that in the SI curve, indicating that the containment and closure policies preceded the decline of human mobility. No significant relations between SI and PI were observed in Chile, South Korea, Sweden, and Japan.

Furthermore, the panel vector autoregression (PVAR) model was used to examine the dynamic relationships between SI, the growth rate of new COVID-19 cases (*D.addcase*), and the policy stringency index (PI) using one week as the time granularity. All three variables pass the unit root test (see Table S4), indicating that the PVAR models can be constructed. According to the optimal lag order test (see Table S5–S6), it can be determined that the optimal lag order of the PVAR models was lag 2 stage. The parameters of the PVAR models were estimated using the generalized method of moments (GMM) (Table 1). The estimation results all passed the stability condition test (see Fig. S2).

**Table 1:** Results of PVAR analysis.

Variables	(1)	(2)	(3)	(4)
	*SI*	*D.addcase*	*SI*	*PI*
L.*SI*	1.2943*** (0.037)	1.1763* (0.678)	1.0792*** (0.060)	0.0371 (0.045)
L2.*SI*	− 0.3424*** (0.037)	− 1.2675* (0.703)	− 0.0202 (0.047)	0.0299 (0.040)
L.*D.addcase*	0.0049*** (0.001)	0.3711*** (0.116)		
L2.*D.addcase*	0.0023** (0.001)	0.2160** (0.089)		
L.*PI*			0.2389** (0.114)	1.3306*** (0.092)
L2.*PI*			− 0.4386*** (0.059)	− 0.4705*** (0.060)
Observations	1088	1088	1122	1122

Standard errors in parentheses, ***P < 0.01, **P < 0.05, *P < 0.1

We found a bidirectional causality relationship between SI and D.addcase. On one hand, the results showed that the increase of D.addcase with lags one and two weeks significantly promoted the current SI increase, indicating that the outbreak of the COVID-19 pandemic has had a huge impact on mobility, economy, and society of the study countries. On the other hand, the increase in SI with one-week lagging had a positive impact on D.addcase in this period, but the increase in SI with two weeks lagging significantly reduced D.addcase in this period, suggesting that the impact of SI on COVID-19 cases has a time lag, that is, it cannot take effect quickly within a week but may need at least two weeks to suppress the COVID-19 pandemic.

Further, a one-way causality relationship between SI and PI was detected. Specifically, increased PI with one-week lagging significantly increased SI in the current period, indicating that the policy implementation can take effect quickly. However, the results of lagging 2 weeks showed that the implementation of travel restrictions and social policies was difficult to remain effective in the long run. Meanwhile, PI did not change with the change of SI in the lag period, indicating that there is a one-way causal relationship between SI and PI.

**Figure 4:**
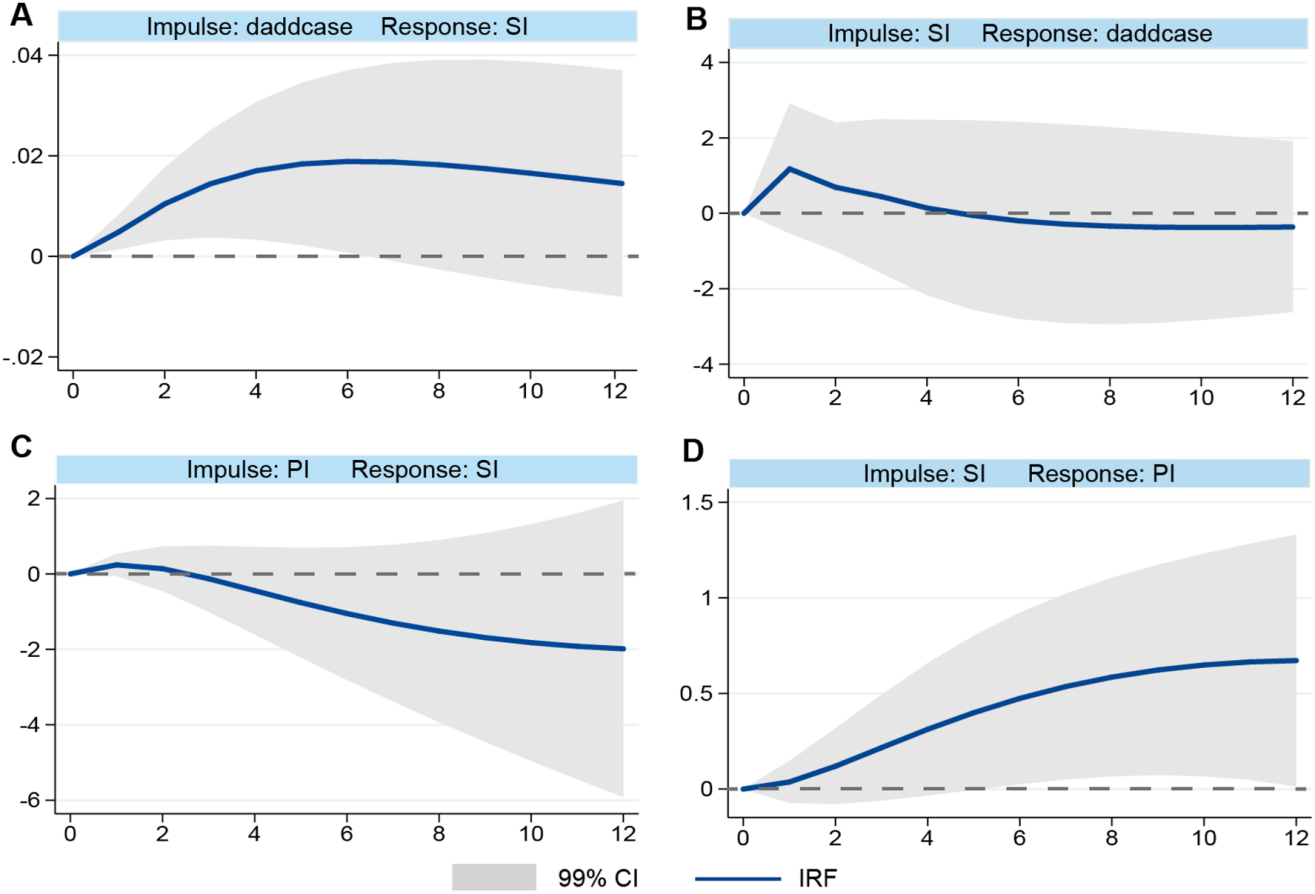
Results of impulse response function (IRF) analysis with silent index (SI), the growth rate of new COVID-19 cases (daddcase), and the policy stringency index (PI). For example, (**A**) indicates the response of SI to daddcase. Impulse response results were obtained by running 500 iterations of the Monte Carlo Simulation; the upper and lower curves represent the 99% CI. The horizontal axis indicates the step numbers of the twelve-period impulse response function.

Moreover, a 12-period (week) impulse response function (IRF) was conducted to reveal the change process of SI responding to D.addcase and PI shocks over a longer period (Fig. 4). First, an inverted U-shape curve was observed for the impact of D.addcase on SI (Fig. 4A); SI increased rapidly in 4 weeks, but then flattened and gradually decreased over time. Second, we observed a lagging response in D.addcase from the impact of SI (Fig. 4B); D.addcase maintained an upward trend first for about a week, and then decreased over time. Third, we observed a slight rising trend of SI due to the impact of PI in about a week, followed by a declining trend afterwards (Fig. 4C), which is consistent with the PVAR results. Last, we observed a positive association between SI and PI with little lagging response (Fig. 4D), which is likely because both reflect the response of public and government to the aggravation of the COVID-19 pandemic, as our PVAR analysis showed no direct significant influence of SI on PI.

### SI and COVID-19 infection rate in different groups of GNI per capita

**Figure 5:**
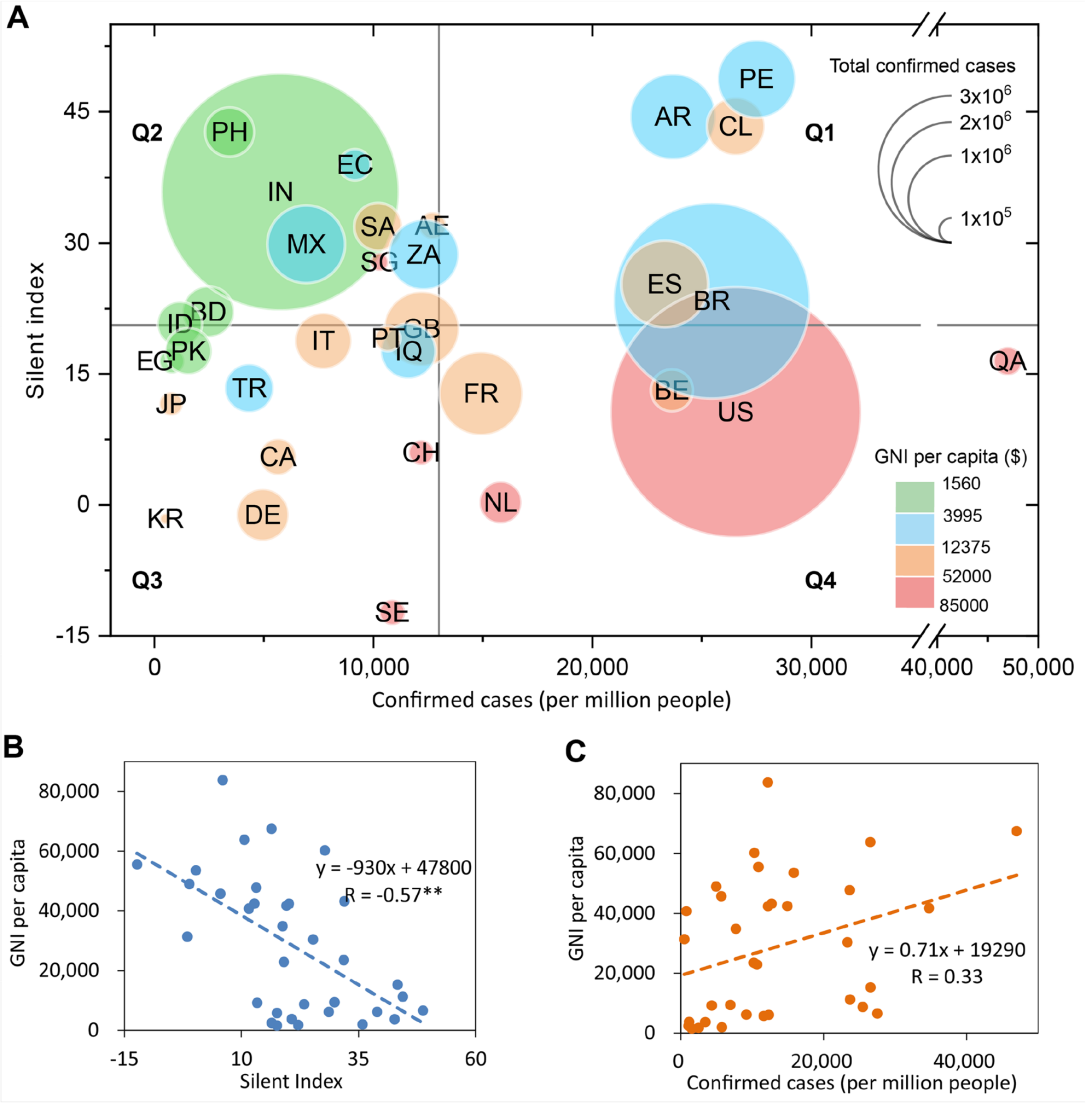
SI and COVID-19 infection rate in different groups of GNI per capita. (**A**) The average SI is calculated from the day with 100 cumulative COVID-19 cases to Oct. 24, 2020. The horizontal gray line represents the average value of the SI, and the vertical gray line represents the average value of the confirmed COVID-19 cases per million population. The classification of GNI per capita is based on the method of the World Bank Atlas. (**B**) The Pearson correlation coefficient (R) between SI and GNI per capita of 33 countries is -0.57, and there is a significant correlation at the 0.01 level. (**C**) Pearson correlation coefficients between COVID-19 infection rate and GNI per capita of 33 countries are not significant.

Further analysis in Fig. 5A showed that SI and COVID-19 infection rates were different in different groups of GNI per capita by Oct. 24, 2020. In general, SI and GNI per capita have a significant negative linear relationship (Fig. 5B), but GNI per capita and COVID-19 infection rate showed a weak and insignificant positive relationship (Fig. 5C). In the quadrantal diagram of SI and standardized COVID-19 cases, most developed countries (including the United States, Switzerland, Sweden, Singapore, Netherlands, and Qatar) were in the Q4 region——high incidence rate and low SI. While less developed countries were mostly in the Q2 region——low incidence rate and high SI (including India, Pakistan, South Africa, Peru, Ecuador, Mexico, Turkey, and Brazil. Belarus is an exception). For the middle-income countries (blue circles), SI was positively correlated with the standardized total COVID-19 cases per million population. During the study period, the economically underdeveloped countries had higher SI, which likely attribute to the mitigation policy and helped these countries to delay the pandemic spread, but also meant their economic system was more affected by the COVID-19 pandemic.

## Discussion

**Figure 6:**
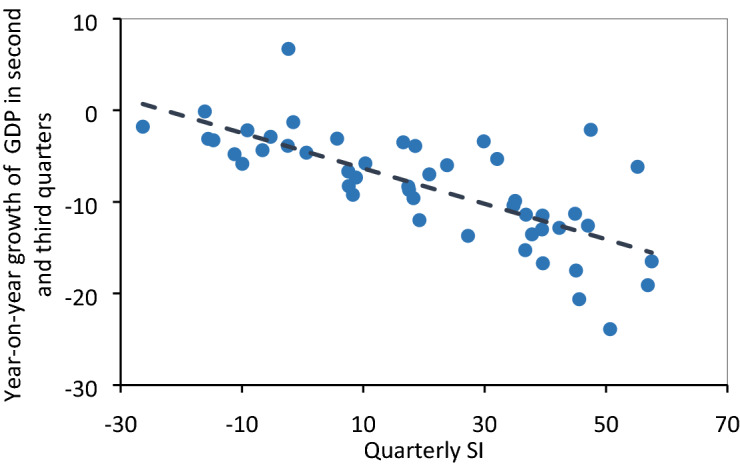
A significant linear relationship between SI and GDP growth quarterly. Each dot represents a country. The regression equation between SI and GDP growth rate quarterly is y = − 0.19x − 4.41, and there was a significant correlation at the 0.01 level. The GDP growth in the second and third quarters can be found in the International Monetary Fund (data.imf.org) and the statistical bureau of different countries.

The occasional quietness of the world can be beneficial to our environment and health^39^; however, the long-term silence has profound impacts on every aspect of the human system, making the achievement of the Sustainable Development Goals (SDGs) even more urgent^40^. While the impact of the pandemic will vary from country to country, it will most likely increase poverty and inequalities on a global scale^41^. A timely assessment of the comprehensive impact of COVID-19 is very important. Thus, we constructed the silent index (SI), a composite measure of daily human mobility across five-place categories and a more intuitive and easy-to-understand indicator of lack of mobility at the population level than the Google Mobility Index (GMI). Meanwhile, we found a significant linear relationship between SI and GDP growth quarterly (Fig. 6), indicating that, to some extent, SI can reflect the disturbing impact of disasters or catastrophic events on the activities related to global or national economy, except for reflecting human mobility. Several studies have evaluated the social and economic losses^42,43^, and estimated the slowdown of GDP for different countries under many scenarios due to the impact of COVID-19 pandemic^44,45^. Different from these studies, SI can be used as a real-time indicator of slowing economic activities and provide timely data to government and policymakers for decision making. For many countries and regions with sufficient Google users, the SI can be applied to reflect relative changes in economic activities in real-time and at a high temporal resolution, which can solve three main problems in traditional economic-statistical indicator data: 1) lack of the data in certain countries/regions, 2) delay in getting the data, and 3) coarse temporal resolution in the data. Thus, the SI be also used as a proxy to quickly and timely monitor the changes in human social-economic activities and help public policy decision making at a global scale.

A case study at the urban scale of Italy showed that mobility contraction is stronger in municipalities where income per capita is lower^6^, which is consistent with our national-scale result In general, the impact of the COVID-19 pandemic on less developed countries and low-income populations is more severe, exacerbating the inequalities. Another study has shown that daily COVID-19 cases were directly related to the mobility habits 21 days before^46^, which agrees well with our result from 33 countries that the impact of mobility on new COVID-19 cases lagged by at least 2 weeks. The COVID-19 incidence in some countries showed that the lower the mean income, the higher the COVID-19 rate^47,48^. However, the analysis based on 33 countries does not show a significant relationship between national income and COVID-19 incidence (Fig. 5C). At the national level, the relationship is not yet conclusive.

The results of this study indicated that travel restrictions and social policies could take effect in one week, however, they are difficult to remain effective or be sustainable in the long run. This might be related to the degree of citizens’ cooperation and government interventions^49^. Strict social distancing policies reduce the severity of the pandemic during the lockdown period, but a full recovery of the contagion can occur once such measures are relaxed^50^. Thus, a big challenge of the COVID-19 is how to balance economic and social activities with pandemic prevention and control^51,52^. Ideally, we would hope to see a gradual decline in both the silent index and the number of new cases in the future. To achieve this goal, we need more intelligent social governance, collaboration on global efforts, especially with regard to the development and distribution of vaccines, food, and anti-epidemic supplies^53^, more effective medical protection measures, more motivated and educated population practicing evidence-based self-protection actions and vaccination, especially in low- and middle-income countries^54,55^.

This study has several limitations. First, we only studied differences at the country level, without considering the heterogeneity within each country. Second, only Google’s mobility data were applied in this paper, thus some countries such as China and North Korea with few Google users cannot be evaluated in this study. In the future, the methodological integration of Google, Apple, Baidu, and other mobile big data can be combined to enlarge the scope and improve the accuracy of SI. Further, the application of the mobility data generated by Internet companies also has some limitations. For instance, whether mobility patterns based on the mobility data can be generalized to the public is debatable^16^. It indicated that the use of this kind of data as a proxy for global mobility dynamics needs further justification. Third, our time-series data does not consider the seasonable variation of human mobility. The limitation of the baseline setting may ignore the human mobility difference among different seasons. Fourth, although the causal relationships have been detected in this study, it mainly indicates the causality in economic modeling such as PVAR. For the causation of COVID-19 spread and independent variables in epidemiology, further investigations need to be considered. Last, although SI reflects economic development, and maybe some of the place-specific mobility indexes capture social activities (e.g., entertainment venue, workplace, and park), we were limited by focusing only on SI to evaluate the impact of COVID. Hence, comprehensive consideration of each country's features and combination with other social-economic big data may further improve the accuracy of the assessment^34^.

In summary, compared to the existing literature, the innovation and scientific contribution of our work is that: First, we estimated the heterogeneity that existed in human mobility reduction across countries and places, and observed a drastic impact of the COVID-19 pandemic on human mobility, which decreased by 12.5% from February to mid-October 2020. Second, the bi-directional dynamic relationships between SI and COVID-19 new cases were detected with a lagging period of 1–2 weeks, indicating that the outbreak of the COVID-19 pandemic has had a huge impact on mobility, economy, and society of the study countries with lags of one and two weeks. Meanwhile, the impact of SI on new COVID-19 cases has a time lag, that is, it may take at least two weeks to suppress the COVID-19 pandemic. Third, a significant linear relationship between SI and GDP growth quarterly, indicating that SI may reflect the disturbing impact of disasters or catastrophic events on the activities related to the global or national economy. Furthermore, it indicated that the travel restrictions and social policies could take effect in one week, however, they are challenging to remain effective in the long run. Finally, we found underdeveloped countries are more affected by the COVID-19 pandemic.

## Methods

### Silent index (SI) construction

We take advantage of the advent of mobile location-based services accessed via smartphones, for which daily data about human mobility are becoming available. In this paper, we collected the Google Mobility Index (GMI) that shows how visits to main place categories change compared to the baseline as a positive or negative percentage at the country level (https://www.google.com/covid19/mobility/). The baseline is the median value, for the corresponding day of the week, during the 5-week period Jan 3–Feb 6, 2020. Since the penetration of smartphones, location accuracy of global positioning system data, and the understanding of categorized places vary from region to region, we selected 33 countries to study the silent index, all of which had high popularity of Google service on mobile phones and experienced a relatively serious COVID-19 pandemic.

In this study, the silent index (SI) was constructed to comprehensively assess the variation of human mobility at the county level based on Google's mobility big data. The name of this index is inspired by *Silent Spring*, written by American marine biologist and conservationist Rachel L. Carson, who described the absence of the sound of birds and insects due to the overutilization of pesticides in the environment^56^. The SI was constructed based on the GMI in five categorized places (grocery & pharmacy, parks, transit stations, retail & recreation, workplaces) with equal weights. These places mainly reflect different urban functions including city vitality, economic performance, and level of pandemic prevention measures (e.g., lockdown and stay-at-home orders), respectively. This SI describes the relative change of daily human mobility compared with that before the COVID-19 pandemic for a specific region. Further, we calculated a global SI by weighting the SI of each region (i.e., a country in this study) by their populations as follows:

1 $$ SI_{t} = - \frac{1}{5}\mathop \sum \limits_{i = 1}^{5} GMI_{it} $$

2 $$ Global\_SI_{t} = \mathop \sum \limits_{j = 1}^{n} (SI_{jt} \times W_{j} ) $$

In Eqs. (1) and (2), *i* presents the different categorized places, *t* is the date, *W*_*j*_ is the proportion of the population of country *j* in the total population of all studied countries, *n* is the number of studied countries and regions which is 126 in this study. Considering the large variation in the SI on weekdays and weekends, and the effect of weather on mobility, we use the weekly average to reflect the smooth changes of a long-term series of SI.

### COVID-19 cases data

In this study, the total confirmed cases and newly confirmed daily COVID-19 cases at the country level were collected from the *WHO Coronavirus Disease (COVID-19) Dashboard* (https://covid19.who.int/table). The time span of our collected dataset ranges from February 15th to middle October 2020 with a total of 36 weeks. The total confirmed number reflects the degree of the impact of COVID-19 in a country, and the newly confirmed cases indicate the spread trend of the COVID-19. To compare with SI, we used the number of cases on the last day of the week for total confirmed COVID-19 cases and the total number of new cases in one week for newly confirmed cases. The baseline day of the COVID-19 pandemic is considered when cumulatively 100 confirmed COVID-19 cases were reported. The country-level population used in the calculation of confirmed COVID-19 cases per million population comes from the World Development Index database 2020 (https://databank.worldbank.org/reports.aspx?source=world-development-indicators).

### Policy stringency index (PI)

Policy stringency index (PI) was designed to measure the government interventions to the COVID-19 spread by combining a series of indices to aggregate various measures (www.bsg.ox.ac.uk/covidtracker)38. PI was created into a composite score between 0 and 100, which included government restrictions and closing of school, workplace, public transport, restrictions on internal and international movement, and other economic, containment, and health measures, etc. (see Table S3). PI was calculated as follows:

3 $$ I_{kt} = 100\frac{{v_{kt} - 0.5\left( {F_{k} - f_{kt} } \right)}}{{N_{k} }} $$

4 $$ PI = \frac{1}{n}\mathop \sum \limits_{k = 1}^{n} I_{k} $$

In Eqs. (3) and (4), *I* means each sub-index score for any given indicator (*k*) on any given day (*t*); *N*_*k*_ is the maximum value of the indicator, whether that indicator has a flag (*F*_*k*_ = 1 if the indicator has a flag variable, or 0 if the indicator does not have a flag variable), the recorded policy value on the ordinal scale (*v*_*kt*_), the recorded binary flag for that indicator, if that indicator has a flag (*f*_*kt*_).

### Hierarchical clustering

We obtained a clustered heatmap using the hierarchical clustering method (Fig. S1) and four clusters are used in our clustering results based on the average silhouette method (Table S1)^57^. The order of the rows is determined by performing hierarchical cluster analyses of the rows, which tend to position similar rows together on the plot. Euclidean distance was applied as the dissimilarity measure and the average-linkage method is used to obtain an average inter-cluster distance. In this study, hierarchical clustering was used to calculate the SI variation from the baseline day to middle October 2020 with the dendrogram in the heatmap of the selected countries. The analysis was performed through the R software with essential package *gplots*^58^*.*

### Panel vector autoregression (PVAR)

#### PVAR modeling

One of the main aims of this study was to investigate the potential bi-directional causal relationship between SI and the growth rate of new COVID-19 cases. The increase of SI may slow down the spread of COVID-19, while the increase in newly confirmed cases may cause an increase in SI. The panel vector autoregression (PVAR) model has been widely applied to examine causal relationships of financial conditions and investment^59^, the impact of renewable energy and financial development on carbon dioxide emissions and economic growth^60^, and the dynamics of mental well-being and other social factors^61^. The PVAR has several advantages. First, it considers the lag periods to reflect the interactive dynamic relationship of each variable; all variables are typically treated as endogenous in the PVAR, which is appropriate for this paper. Second, by estimating the PVAR coefficients, it not only determines the positive and negative effects of the explaining variables, but also reflects the magnitude of the impact. Third, the PVAR model allows the individual effect and heteroscedasticity in the data. Due to the existence of many cross-section data, the model allows the lag coefficient to change with time, which relaxes the requirement of temporal stability of the data. Therefore, in this study, general methods of the moment were applied in the PVAR to detect the dynamic relationship between SI and the growth rate of new COVID-19 cases.

The PVAR models can be constructed as the following. Among them, models (5) and (6) were constructed to reveal the direct effect of SI on suppressing the growth rate of new COVID-19 cases, and models (7) and (8) were constructed to probe the relationship between SI and PI.

5 $$ D.addcase_{j,t} = w_{0} + \mathop \sum \limits_{l = 1}^{n} \alpha_{l} \;D.addcase_{j,t - l} + \mathop \sum \limits_{l = 1}^{n} \beta_{l}\; SI_{j,t - l} + f_{j} + \varphi_{t} + u_{jt} $$

6 $$ SI_{j,t} = \varepsilon_{0} + \mathop \sum \limits_{l = 1}^{n} \gamma_{l}\; SI_{j,t - l} + \mathop \sum \limits_{l = 1}^{n} \theta_{l} \;D.addcase_{j,t - l} + f_{j} + \varphi_{t} + u_{jt} $$

7 $$ PI_{j,t} = w_{0} + \mathop \sum \limits_{l = 1}^{n} \alpha_{l}\; PI_{j,t - l} + \mathop \sum \limits_{l = 1}^{n} \beta_{l} \;SI_{j,t - l} + f_{j} + \varphi_{t} + u_{jt} $$

8 $$ SI_{j,t} = \varepsilon_{0} + \mathop \sum \limits_{l = 1}^{n} \gamma_{l} \;SI_{j,t - l} + \mathop \sum \limits_{l = 1}^{n} \theta_{l}\; PI_{j,t - l} + f_{j} + \varphi_{t} + u_{jt} $$

In the above equations, *D.addcase*_*j,t-1*_, *SI *_*j,t-1*_, and *PI *_*j,t-1*_ indicated the explanatory variables based on the *l*-order lag period of the growth rate of new COVID-19 cases per million people per week (i.e., the first-order difference of newly confirmed cases), the silent index, and the policy stringency index (PI). Meanwhile, *α*_*l*_*, γ*_*l*_*, β*_*l*_ and *θ*_*l*_ were the estimated coefficients of each lagged explanatory variable. *f*_*j*_ represented the individual fixed effect in PVAR modeling to consider the heterogeneity across the country.$\varphi_{t}$ represented the time fixed effect. *u*_*jt*_ represented the random disturbance term, and *w*_0_ and *ε*_0_ were the intercepts. The subscripts *l*, *j,* and *t* represented the number of lag periods, different countries, and dates, respectively. Combining the three criteria of MBIC, MAIC, and MQIC, we found that the optimal lag orders of the two sets of PVAR models are both lagging by 2 periods (see Table S5–S6).

#### Unit root test

Additionally, before the PVAR, the unit root test was conducted to avoid false regression^62^. In this study, two widely used panel unit test methods were adopted, namely the LLC test for common root tests and the IPS test and the Fisher-ADF test for heterogeneous unit root tests. The test results were shown in Table S4. All three variables passed the unit root test, indicating the stationarity of these panel data.

### Impulse response function (IRF)

The impulse response functions (IRF) were derived from the estimated quadrivariate VAR models by using the Cholesky decomposition method^63^. This method can describe the evolution of a model’s variables in reaction to a shock in one or more variables, and this feature allows us to trace the transmission of a single shock within an otherwise noisy system of equations. Hence, in this study, the IRF depicts the changing trend of the impact of the change of SI on the growth rate of new COVID-19 cases, the changing trend of the impact of the growth rate of new COVIS-19 cases on SI, and the dynamic relationship between SI and policy stringency index.

## Supplementary Information

Supplementary Information.

## Data Availability

All input data used in these analyses were derived from published sources cited in the Methods. Any other datasets generated in the current study are available from the corresponding author upon request.
