# Supplementary material for: Observing the silent world under COVID-19 with a comprehensive impact analysis based on human mobility
Source: Sci Rep. 2021 Jul 19;11:14691. doi: 10.1038/s41598-021-94060-4 (PMC8289815; doi:10.1038/s41598-021-94060-4)
Supplement: Supplementary file 1 — Supplementary Information. [file 41598_2021_94060_MOESM1_ESM.docx]

**Observing the silent world under COVID-19 with a comprehensive impact analysis based on human mobility**

**Shaobin Wang^1^, Yun Tong^2^, Yupeng Fan^1^, Haimeng Liu^1*^,** **Jun Wu^3^, Zheye Wang^4^, Chuanglin Fang^1^**

1 Institute of Geographic Sciences and Natural Resources Research, Chinese Academy of Sciences,

Beijing, China

2 School of Tourism, Hainan University, Haikou, China

3 Program in Public Health, Susan and Henry Samueli College of Health Sciences, University of California, Irvine, USA

4 Kinder Institute for Urban Research, Rice University, Houston, USA

* Corresponding author

Email: [liuhm@igsnrr.ac.cn](mailto:liuhm@igsnrr.ac.cn)

#### Supplemental Information

#### Supplemental Figures


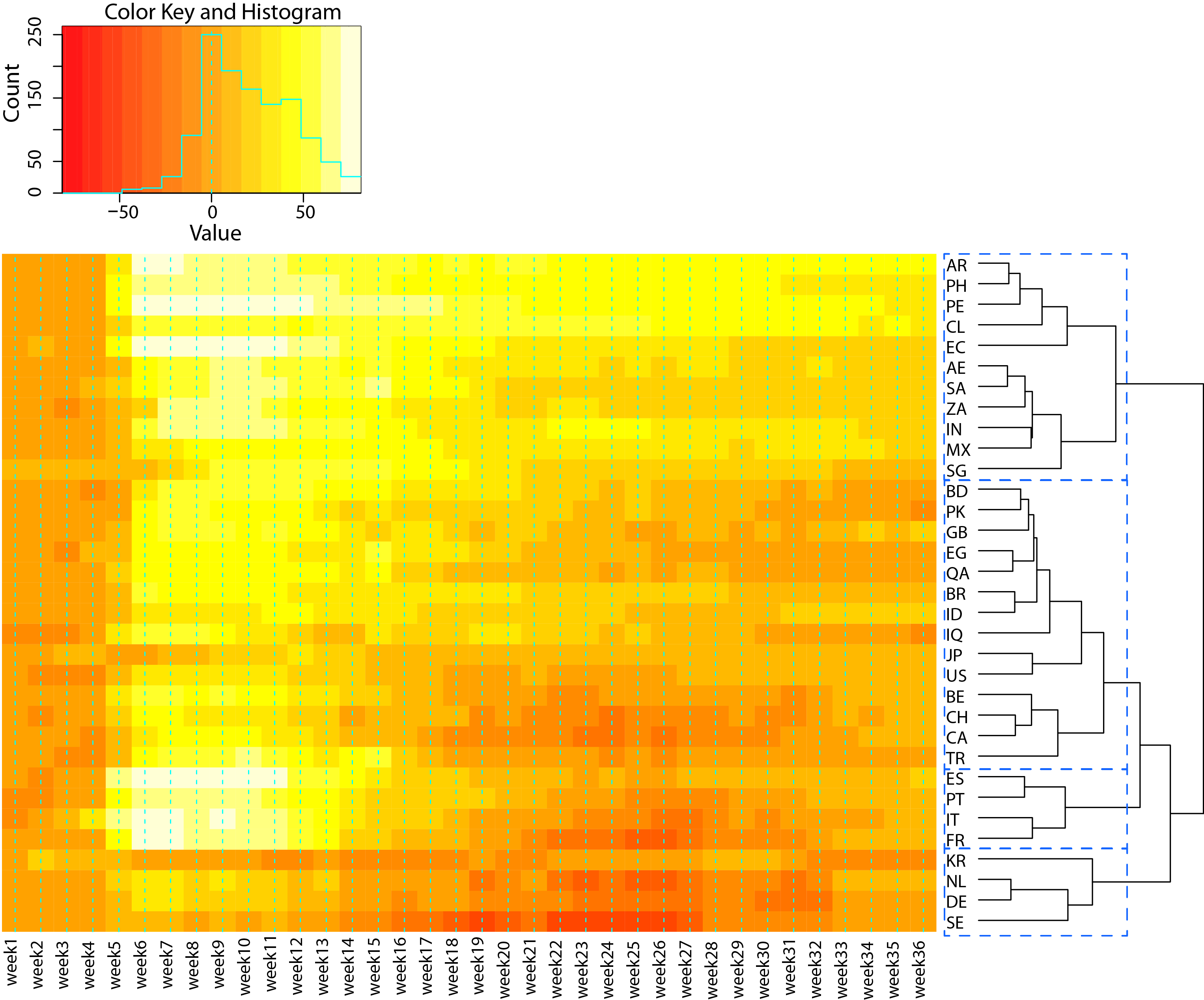


**Figure S1. Hierarchical clustering results of SI variation from the baseline day to middle October 2020 based on the dendrogram in the heatmap of 33 selected countries.** The order of the rows is determined by performing hierarchical cluster analyses of the rows, which tend to position similar rows together on the plot. Four clusters are used in our clustering results based on the average silhouette method.


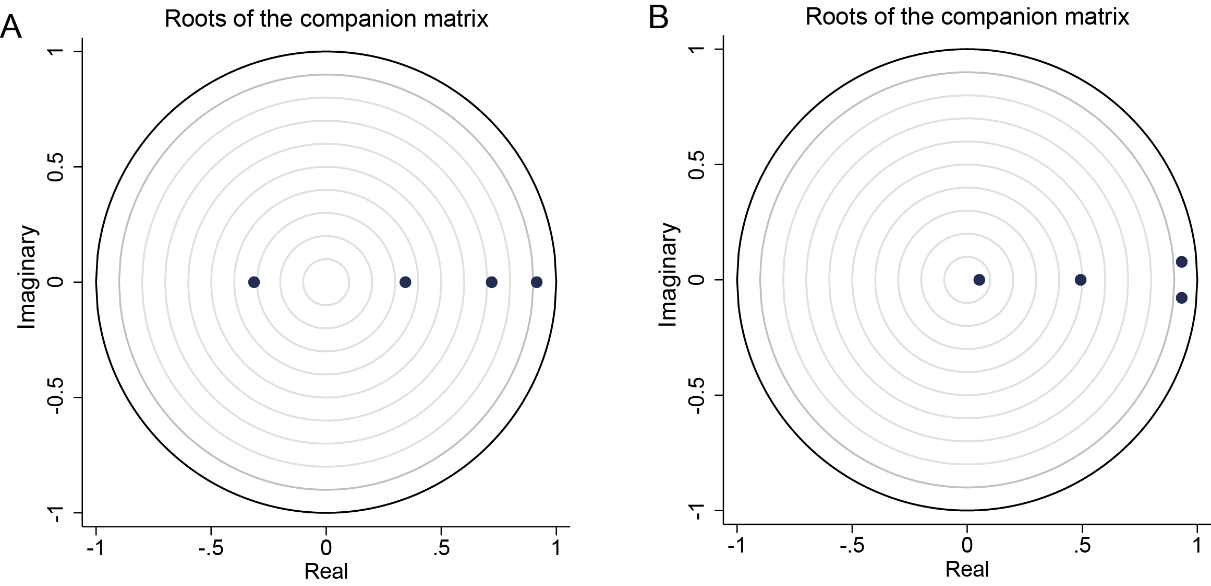


**Figure S2.** Stability condition test. The black points represent the reciprocal of the root of the AR characteristic polynomial. All points in the stability test diagram of the two PVAR models are distributed in the unit circle, that is, the reciprocal of the roots of all characteristic polynomials is less than 1, indicating a good model fitting.

#### Supplemental Tables

**Table S1. Silhouette scores of different numbers of clusters**

| Number of clusters | Silhouette score |
| --- | --- |
| 2 | 0.3 |
| 3 | 0.4 |
| 4 | 0.5 |
| 5 | 0.3 |
| 6 | 0.2 |

**Table S2. Abbreviations of countries and regions in this study**

| Country code | Country |
| --- | --- |
| AE | United Arab Emirates |
| AT | Austria |
| BD | Bangladesh |
| BE | Belgium |
| BR | Brazil |
| BY | Belarus |
| CA | Canada |
| CH | Switzerland |
| CL | Chile |
| DE | Germany |
| EC | Ecuador |
| EG | Egypt |
| ES | Spain |
| FR | France |
| GB | the United Kingdom |
| IE | Ireland |
| IN | India |
| IT | Italy |
| JP | Japan |
| KR | South Korea |
| MX | Mexico |
| NL | Netherlands |
| PE | Peru |
| PK | Pakistan |
| PT | Portugal |
| QA | Qatar |
| SA | Saudi Arabia |
| SE | Sweden |
| SG | Singapore |
| TR | Turkey |
| US | United States |
| ZA | South Africa |

**Table S3. Indicators and flag values of policy stringency index**

| Indicator | Max value (N_k_) | Flag? (F_k_) |
| --- | --- | --- |
| School closing (C1) | 3 (0, 1, 2, 3) | Yes=1 |
| Workplace closing (C2) | 3 (0, 1, 2, 3) | Yes=1 |
| Cancel public events (C3) | 2 (0, 1, 2) | Yes=1 |
| Restrictions on gathering size (C4) | 4 (0, 1, 2, 3, 4) | Yes=1 |
| Close public transport (C5) | 2 (0, 1, 2) | Yes=1 |
| Stay at home requirements (C6) | 3 (0, 1, 2, 3) | Yes=1 |
| Restrictions on internal movement (C7) | 2 (0, 1, 2) | Yes=1 |
| Restrictions on international travel (C8) | 4 (0, 1, 2, 3, 4) | No=0 |
| Public information campaign (H1) | 2 (0, 1, 2) | Yes=1 |

The coding of each indicator (0-4) can be found in the OxCGRT documentation ([www.bsg.ox.ac.uk/covidtracker](http://www.bsg.ox.ac.uk/covidtracker)).

| **Table S4. Unit root test result** | | | | |
| --- | --- | --- | --- | --- |
|  | LLC | | IPS | |
| Variable | Statistics | P-Value | Statistics | P-Value |
| *D.addcase* | -4.3063*** | 0.0000 | -10.0355*** | 0.0000 |
| *SI* | -3.7652*** | 0.0001 | -1.2918* | 0.0982 |
| *PI* | -10.6877*** | 0.0000 | -7.9558*** | 0.0000 |

*** p<0.01, ** p<0.05, * p<0.1

**Table S5. Lag-order selection statistics between SI and COVID-19 cases**

| **Lag** | **MBIC** | **MAIC** | **MQIC** |
| --- | --- | --- | --- |
| 1 | -120.0545* | 13.91033 | -37.30919 |
| 2 | -111.5786 | 3.248379* | -40.65407* |
| 3 | -87.85461 | 7.834534 | -28.75084 |
| 4 | -71.52466 | 5.026655 | -24.24164 |

* Indicates that the criterion which lags order is determined. We find two of the three test indicators reveal an optimal lag order of 2.

**Table S6. Lag-order selection statistics between SI and PI**

| **Lag** | **MBIC** | **MAIC** | **MQIC** |
| --- | --- | --- | --- |
| 1 | -93.14911 | 41.87242 | -9.657751 |
| 2 | -104.1114* | 11.62135 | -32.54737* |
| 3 | -84.60945 | 11.8345 | -24.97277 |
| 4 | -75.53623 | 1.618925* | -27.82689 |

* Indicates that the criterion which lags order is determined. We find two of the three test indicators reveal an optimal lag order of 2.
